# Study on Durability and Pore Characteristics of Concrete under Salt Freezing Environment

**DOI:** 10.3390/ma14237228

**Published:** 2021-11-26

**Authors:** Xinchao Zheng, Fang Liu, Tao Luo, Yanfu Duan, Yu Yi, Cheng Hua

**Affiliations:** 1 School of Civil Engineering, Xijing University, Xi’an 710123, China; zxcsjy1028@163.com (X.Z.); luotao@xijing.edu.cn (T.L.); 20210117@xijing.edu.cn (Y.D.); yiyu1105@163.com (Y.Y.); huacheng19970609@163.com (C.H.); 2 Shaanxi Key Laboratory of Safety and Durability of Concrete Structures, Xi’an 710123, China

**Keywords:** industrial computed tomography, salt freezing, pore characteristics, compressive strength, concrete durability

## Abstract

The macroscopic mechanical properties and frost resistance durability of concrete are closely related to the changes in the internal pore structure. In this study, the two-dimensional and three-dimensional ICT (Industrial Computerized Tomography) pore characteristics of C30 concrete specimens before and after freezing and thawing in clean water, 5 wt.% NaCl, 5 wt.% CaCl_2_, and 5 wt.% CH_3_COOK solution environments are obtained through concrete frost resistance durability test and ICT scanning technology. The effects of pore structure changes on concrete frost resistance, durability, and compressive strength mechanical properties after freezing and thawing cycles in different salt solution environments are analyzed. This paper provides new means and ideas for the study of concrete pores. The results show that with the increase in the freezing and thawing times, the concrete porosity, two-dimensional pore area, three-dimensional pore volume, and pore number generally increase in any solution environment, resulting in the loss of concrete compressive strength, mortar spalling, and the decrease in the relative dynamic elastic modulus. Among them, the CH_3_COOK solution has the least influence on the concrete pore changes; the NaCl solution has the greatest influence on the change in the concrete internal porosity. The damage of CaCl_2_ solution to concrete is second only to the NaCl solution, followed by clean water. The increase in the concrete internal porosity from high to low is NaCl, CaCl_2_, clean water, and CH_3_COOK. The change in the pore volume of 0.1 to 1 mm^3^ after the freeze–thaw cycle is the main factor for reducing concrete strength. The test results have certain guiding value for the selection of deicing salt in engineering.

## 1. Introduction

Deicing salt can effectively reduce the freezing point and realize the effect of melting ice and snow to alleviate the traffic pressure caused by snow in winter. However, winter significantly impacts concrete with the melted ice and snow entering the concrete. In Northern China, several deicing salts are used to ensure traffic because the temperature is lower than 0 °C in winter, resulting in peeling and cracks in concrete structures, such as in roads and bridges, which affect their frost resistance durability. Additionally, the concrete strength of some foundation concrete structures, such as dams and bridge foundations, has been greatly affected [1,2,3,4,5]. Therefore, the freeze–thaw damage of concrete in the deicing salt solution environment cannot be ignored.

Currently, there is no unified academic recognition for the salt freezing failure mechanism of concrete. Recently, hydrostatic pressure, osmotic pressure, and temperature stress hypothesis have been more common [6,7,8]. Thus, many scholars have conducted in-depth research on concrete salt freezing damage.

Harnik A.B. [9] believed that the frost resistance durability of concrete is related to different factors. Among them, the most harmful is the increase in the water absorption saturation of concrete due to the presence of salt solution and the increase in the pore osmotic pressure of concrete due to super cooling of the aqueous solution. Yang Q.B. [10,11] believed that the saturation degree of concrete in the deicing salt environment is greater than that in the clean water environment. Therefore, under the freezing–thawing cycle, the increase in concrete pores accelerates the deterioration of pore structure. Some scholars have studied the durability of concrete in different salt-freeze environments. Zheng K. [12] investigated the effect of CaCl_2_, CaAc_2_, and CaFm_2_ solutions on the durability of concrete by the freezing and thawing cycle. The test results show that the mechanical properties of concrete significantly decrease with the increase in the freezing and thawing cycle. They believed that the change in the micro-structure of the salt freezing environment is the main factor for the decrease in the mechanical properties of concrete. Lai Y. [13] investigated the influence of runway deicing salts (the main component is CH_3_COOK) on the salt-freezing resistance of airport runway concrete. They discussed the influence of freezing–thawing cycles in the water and runway deicing salt environment on the apparent morphology, mass loss rate, and relative dynamic elastic modulus of concrete. It is considered that the presence of deicing salts negatively affects the frost resistance of concrete. Cao R.S. [14], employing the freezing method of the concrete freeze–thaw cycle test, studied different cycles, different concentrations, different deicing salt effects on the properties of concrete, concrete in deicing salt NaCl, and CaCl_2_ in a freeze–thaw environment. With the increase in the number of freezing and thawing, the block surface strip material corrosion becomes severe, the dynamic elastic modulus falls, the mechanical properties deteriorate, and the compressive strength decreases. Among them, the test block with a low concentration salt freeze is the most severely damaged. Related personnel [15,16,17,18,19,20] investigated the chemical erosion and depth of deicing salt on concrete. There is a chemical attack on concrete in the deicing salt solution, primarily due to the chemical reaction with the C–S–H gel structure of concrete, resulting in the change in pores in the concrete, which affects the durability and strength of concrete. Mercury intrusion is currently a more popular method of porosity measurement. The mercury intrusion method is often used in materials science and engineering to detect the porosity of concrete, mortar, etc., and to characterize indicators such as pores inside concrete [21,22]. However, the mercury intrusion method has some disadvantages, such as the need to pressurize during the measurement, which will more or less affect the pores of the concrete. Moreover, the sample volume measured by this method is generally small, and it is difficult to represent all of the material characteristics [23].

Several studies on concrete damaged by salt freeze mainly focus on durability life predictions of concrete and mechanical performance and the influence of different mineral contents on concrete frost resistance [24,25,26,27,28,29,30,31,32] to investigate the change in concrete properties after salt and frost damage. Some researchers have used CT scanning technology to show the micro-structure of the concrete [33,34,35,36,37,38]. They discussed the application prospect of CT scanning technology in the concrete pore and two-dimensional (2D) scanning section. However, there is a lack of research on the influence of the change in internal pore characteristics of concrete after salt freezing with different deicing salt solutions on its working performance. Therefore, this paper selects three common deicing salts, NaCl, CaCl_2_, and CH_3_COOK, in the market for durability tests. Additionally, the internal pore characteristics of concrete under different salt freezing environments based on ICT scanning technology are extracted and analyzed. Furthermore, the relationship between pore characteristics and concrete working performance under different salt freezing environments is investigated to further study concrete damage mechanisms by salt freezing.

## 2. Test Scheme

### 2.1. Test Materials

The cement used is conch brand P·O 42.5 ordinary Portland cement.

The Grade I fly ash is produced by Shaanxi Bahe Power Plant.

Coarse aggregate is the nominal size of 5 to 20 mm limestone, with an apparent density of 2835 kg/m^3^ and a bulk density of 1720 kg/m^3^.

Fine aggregate is the Ba River sand in Xi’an, with a fineness modulus of 2.8, an apparent density of 2630 kg/m^3^, and a bulk density of 1480 kg/m^3^.

The admixture is a polycarboxylic acid water-reducing agent.

Ordinary Xi’an tap water is used to prepare the sample. Distilled water is used to prepare the salt solution and soaking.

The salt solutions are 5% NaCl, 5% CaCl_2_, and 5% CH_3_COOK solution. The preparation of the solution is carried out at room temperature.

**Figure 1:**
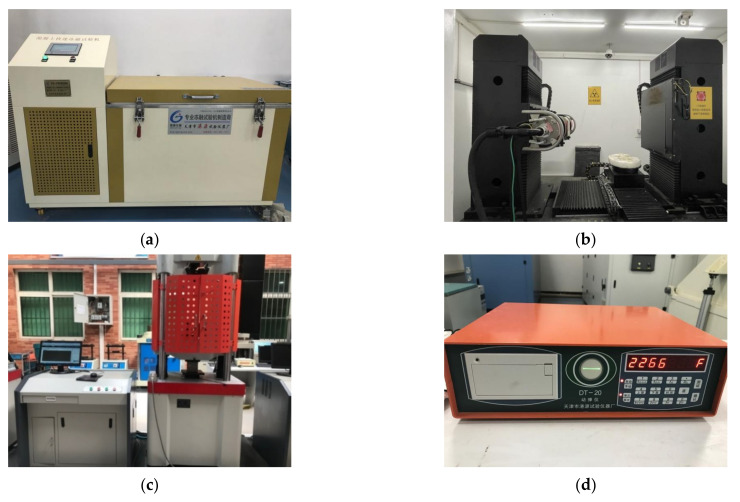
Test instruments: (**a**) TDR-28 Freeze–thaw test chamber; (**b**) MultiscaleVoxel-450 Industrial CT; (**c**) Electro-hydraulic servo universal testing machine; (**d**) DT-20 Dynamic elastic modulus tester.

Instruments and equipment: TDR-28 concrete rapid freezing-thawing testing machine (Gangyuan Test Instrument Factory Tianjin, China), MultiScalevoxel-450 Industrial CT (Sanying Precision, Tianjin, China), microcomputer-controlled electro-hydraulic servo universal testing machine (Chuanbai Instrument Equipment, Jinan, China), DT-20 dynamometer (Gangyuan Test Instrument Factory, Tianjin, China), as shown in Figure 1.

### 2.2. Test Scheme and Concrete Durability Processing

The freeze–thaw cycle test was conducted according to the quick freezing method in Chinese standard specification GB/T50082-2009 [39]. The concrete compressive strength test was conducted according to GB/T50081-2016 [40].

The time of each freezing and thawing cycle is 2–4 h, and the time for thawing is not less than one-fourth of the entire freezing and thawing time. During the freeze–thaw cycle, the lowest and highest temperature of the specimen center is (−18 ± 2) °C and (5 ± 2) °C respectively.

The fabrication design strength is C30, and the test cube concrete specimen size is 100 mm × 100 mm × 100 mm. Table 1 shows the concrete mix ratio. The concrete specimen was cast in the cubic mold to be stored in the laboratory for 24 h at room temperature. Then, the demolded specimen was cured in a humid chamber (RH 95%) at 20 °C for 28 days to ensure adequate hydration degree.

Concrete specimens are divided into ordinary and ICT groups, a total of 81 pieces. A compressive strength test is conducted for the ordinary group after curing. Additionally, its quality and transverse base frequency data are measured at room temperature after soaking in clean water for 4 d. Then, it is placed in clear water, 5 wt.% NaCl solution, 5 wt.% CaCl_2_ solution, and 5 wt.% CH_3_COOK solution for the freeze–thaw cycle test. After every 25 freeze–thaw cycles, the corresponding specimens are taken out for compressive strength, mass loss rate, and relative dynamic elastic modulus tests.

By detecting the transverse fundamental frequency of concrete, the relative dynamic elastic modulus can be calculated according to the following formula:

(1) $$P_{i}=\frac{f_{ni}^{2}}{f_{0i}^{2}}\times 100$$

In Formula (1), *P*_i_ (%) is the relative dynamic elastic modulus of the *i*-th concrete specimen after n freeze–thaw cycles, *f_ni_* (Hz) is Transverse fundamental frequency of the *i*-th concrete specimen after *n* freeze–thaw cycles, and *f*_0*i*_ (Hz) is initial value of transverse fundamental frequency of the *i*-th concrete before the freeze-thaw cycle.

The relative dynamic elastic modulus *P* takes the arithmetic average of the test results of three specimens as the measured value. When the difference between the maximum value, the minimum value, and the median value exceeds 15% of the median value, this value is invalid, and the remaining data are calculated and averaged. The value is used as the measured value.

The mass loss rate of concrete specimens is calculated by the following formulas:

(2) $$\Delta W_{ni}=\frac{W_{0i}-W_{ni}}{W_{0i}}\times 100$$

(3) $$\Delta W_{n}=\frac{\sum_{i=1}^{3}\Delta W_{ni}}{3}\times 100$$

In Formulas (2) and (3), $\Delta W_{ni}$ (%) is the mass loss rate of the *i*-th concrete specimen after N freeze–thaw cycles. $W_{0i}$ (g) is the mass of the *i*-th concrete specimen before the freeze–thaw cycle test, $W_{ni}$ (g) is the mass of the *i*-th concrete specimen after n freeze–thaw cycles. $\Delta W_{n}$ is the average mass loss rate of a group of concrete specimens after n freeze–thaw cycles.

The mass loss rate is based on the arithmetic average of three data as the measured value. When the difference between the maximum value, the minimum value and the median value exceeds 1% of the median value, the value is invalid, and the arithmetic average of the remaining data is taken as the measured value.

### 2.3. The Data Processing of ICT

The ICT group underwent CT scanning before freezing and thawing after soaking in the corresponding solution after maintenance, followed by the freeze–thaw cycle test and ICT scanning immediately after 25 cycles of freezing and thawing and 50 cycles of freezing and thawing. Then, continued the freeze–thaw cycle test to reduce the residence time of the specimen at room temperature and reduce the experimental error. Since the concrete specimen is severely damaged after 75 freeze–thaw cycles and cannot support its subsequent scanning, the ICT scanning data are recorded to the 50th freeze–thaw cycle.

**Figure 2:**
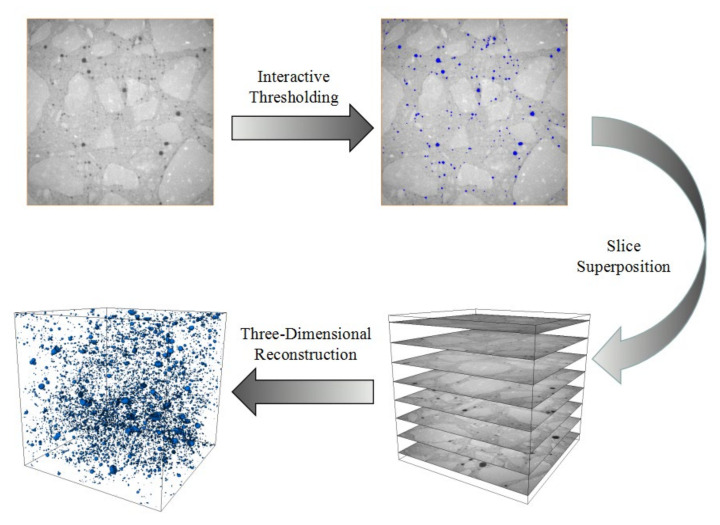
Data processing of ICT.

ICT data processing software is 2 Avizo (2019.1) of ThermoFisher Scientific, Shanghai, China, and the data processing process is shown in Figure 2. After ICT scanning, 2D concrete sections along the height direction were obtained, and stones, mortar, and pores are distinguished according to different gray levels. Among them, the aggregate density is large, showing a bright area, whereas the pore density is small, showing a black area. The pores in the 2D slice of concrete pores are extracted using the interactive threshold segmentation algorithm according to the gray level. The three-dimensional (3D) pore data and concrete model are obtained by stacking the concrete height direction slices.

## 3. Test Results and Analysis

### 3.1. Effects of Different Salt Solutions on Frost Resistance Durability and Mechanical Properties of Concrete

The essential parameters are the compressive strength, mass loss rate, and relative dynamic elastic modulus of concrete to measure its frost resistance durability. Among them, the changes in the mass loss rate and the relative dynamic elastic modulus reflect the intuitive state of concrete, and the change in the compressive strength reflects the strength loss of concrete after the freeze-thaw cycles.

**Figure 3:**
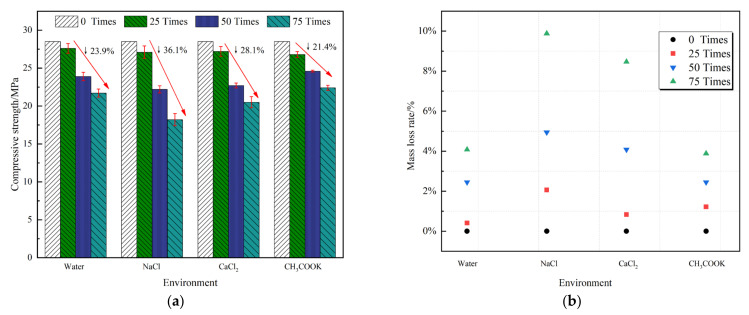

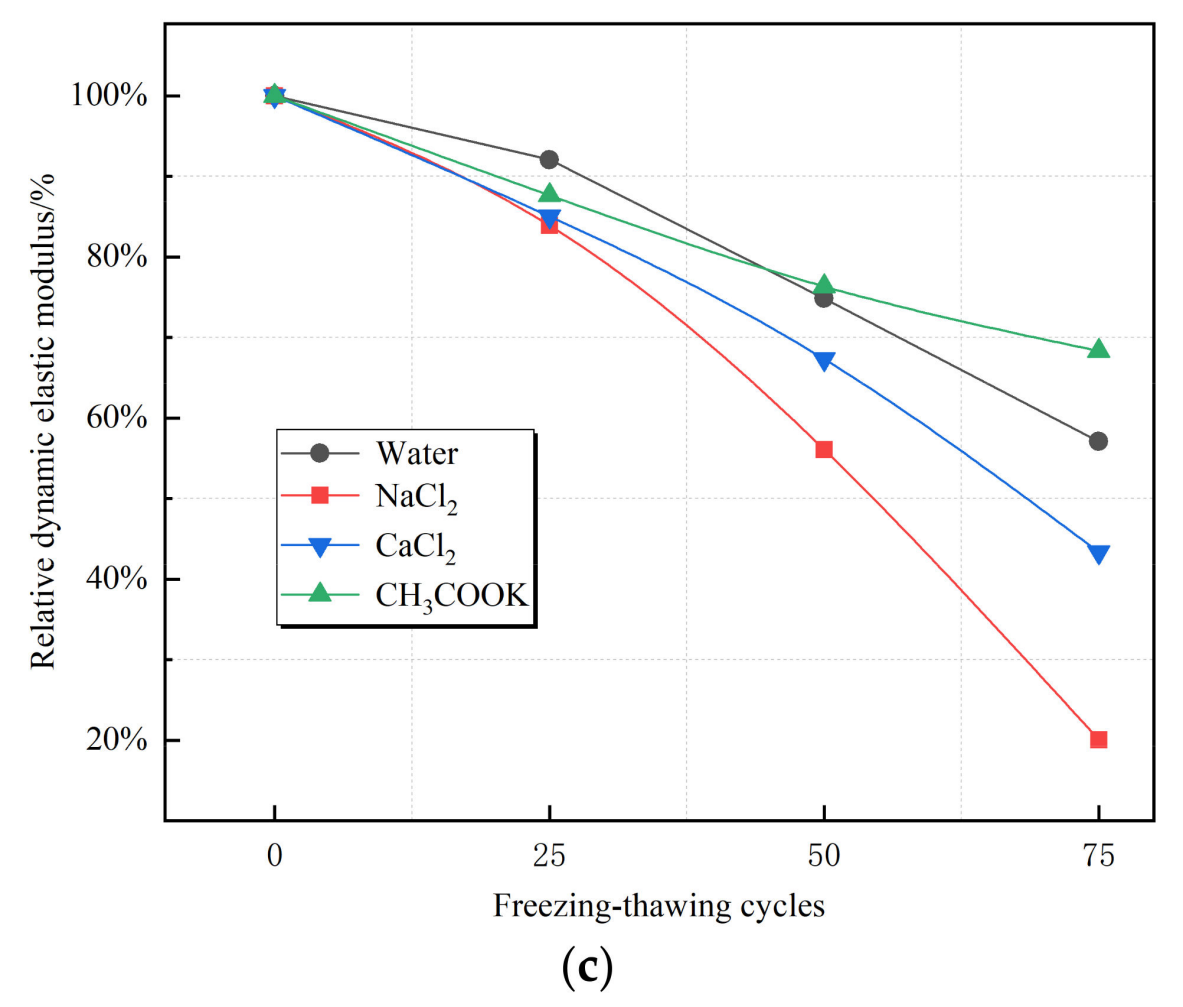
Changes in compressive strength (**a**), mass loss rate (**b**) and relative dynamic elastic modulus (**c**) of concrete under different salt freezing environments.

Figure 3 shows that the mass loss of concrete under the action of freeze–thaw cycles in various solution environments is positively correlated with the number of freeze-thaw cycles. It means that the mass loss of concrete increases as the number of freeze-thaw cycles increases. The relative dynamic elastic modulus and compressive strength are negatively correlated with the number of freeze-thaw cycles. It means that the compressive strength and relative dynamic elastic modulus decrease to a certain extent as the number of freeze-thaw cycles increases.

Figure 3a shows that the compressive strength of the specimen in NaCl solution decreases the most after 75 freeze-thaw cycles, which was 36.1% lower than that before freezing and thawing. The compressive strength of concrete in the CaCl_2_ solution was reduced by 28.1%. The value was 23.9% in a clear water environment. The CH_3_COOK solution environment has little effect on the compressive strength of concrete, and the compressive strength decreases by 21.4% after 75 freeze-thaw cycles.

Figure 3b shows the mass loss rate of concrete under different freeze-thaw cycles. The greater the loss rate, the greater the amount of concrete spalling, and the more obvious the damage caused by salt freezing. Among them, the mass loss rate of concrete in the NaCl solution environment is the largest, which increased by 9.88% after 75 freeze-thaw cycles compared with that before freezing and thawing. Then, followed by the CaCl_2_ solution environment, the mass loss rate of concrete after 75 freeze–thaw cycles was 8.47%. The water and CH_3_COOK solutions have minor effects on the mass loss rate of concrete. After 75 freeze-thaw cycles, the mass loss rates of the water and CH_3_COOK solutions were 4.08% and 3.88%, respectively.

The dynamic elastic modulus is one of the important indices of material mechanical properties. It shows the difficulty of elastic deformation of the concrete test block. The lower the relative dynamic elastic modulus of the concrete test block, the worse its strength, the more severe the damage, and the greater the impact of the freeze–thaw cycle. The durability of concrete is related to the resilience index to some extent. When the concrete is affected by the external environment, it is bound to affect its durability. The worse the durability, the lower the expected resilience [41]. Figure 3c shows that as the number of freeze-thaw cycles increases, at the beginning of the freeze-thaw cycle, the relative dynamic elastic modulus of the concrete test blocks in different environments changes slightly, only attenuates slightly, and the attenuation was close.

After 50 freeze–thaw cycles, the relative dynamic elastic modulus of concrete under the freeze-thaw cycles of NaCl and CaCl_2_ solutions started decreasing rapidly. Among them, the relative dynamic elastic modulus of concrete specimens in the NaCl solution environment decreased significantly faster than that of other specimens, and the attenuation rate continued to accelerate. During the first 50 freeze-thaw cycles, the attenuation of the relative dynamic elastic modulus of the test block in the clean water and CH_3_COOK solutions was the same. However, with the progress of the freeze-thaw cycle, the attenuation rate of the relative dynamic elastic modulus of the test block in the clean water environment begins to be significantly higher than that in the CH_3_COOK solution. After 75 freeze-thaw cycles, the relative dynamic elastic modulus of concrete specimens in clean water, the NaCl solution, the CaCl_2_ solution, and the CH_3_COOK solution were 57.08%, 20.06%, 43.36%, and 68.32%, respectively. Therefore, the attenuation rate of relative dynamic elastic modulus of concrete under four different freeze-thaw cycle media is as follows: CH_3_COOK < Clean water < CaCl_2_ < NaCl.

Through the concrete quality loss tests, the relative dynamic elastic modulus, mechanical properties, and freeze–thaw damage degree of concrete in the clean water environment and 5 wt.% NaCl, 5 wt.% CaCl_2_, and 5 wt.% CH_3_COOK solutions were analyzed. It is consistent with the expected results. In the NaCl solution environment, the concrete quality loss is the most, and mortar peeling is the most severe. After 75 freeze–thaw cycles, the relative dynamic elastic modulus decreased by 79.94%, lower than the limit of 60% in the specification. Additionally, the compressive strength decreased by 36.1% compared with that before freezing and thawing. It can be considered that the freeze-thaw failure has been reached due to the increase in water saturation and the water absorption rate of capillary in concrete because of the existence of the salt solution. The ice pressure generated when the solution freezes will cause more severe damage to the concrete than in the clean water environment [42,43]. The CaCl_2_ solution is acidic, and it has a certain chemical erosion on concrete. In freezing–thawing cycles, it can promote the dissolution of Ca(OH)_2_ in concrete and [44,45] the decomposition of C–S–H gel. After testing, the freezing and thawing damage of concrete in the 5 wt.% CaCl_2_ solution environment is lighter than that in the 5 wt.% NaCl environment. However, the influence of salt freezing damage on coagulation soil cannot be ignored. After 75 freeze-thaw cycles, the mass loss rate reaches 8.47%. The strength decreased by 28.1%, reaching the failure standard. Compared with chloride deicing salts, the CH_3_COOK solution is alkaline while reducing the freezing point, indicating less chemical erosion on concrete. After 75 freeze-thaw cycles, all standards fail to meet the damage conditions in the specification, and it has less impact on the salt freeze–thaw damage of concrete [46].

### 3.2. Two-Dimensional Pore Structure Characteristics

ICT scanning data reconstruction was performed every 25 freeze-thaw cycles to obtain 1000 cross-sectional slices. By comparing the slicing data, the concrete in different freezing and thawing environments along the direction of the casting surface height of each pore distribution of the calculated porosity of variance was obtained, and the average porosity change was further determined to influence the degree of the solution of the concrete’s internal porosity change.

**Figure 4:**
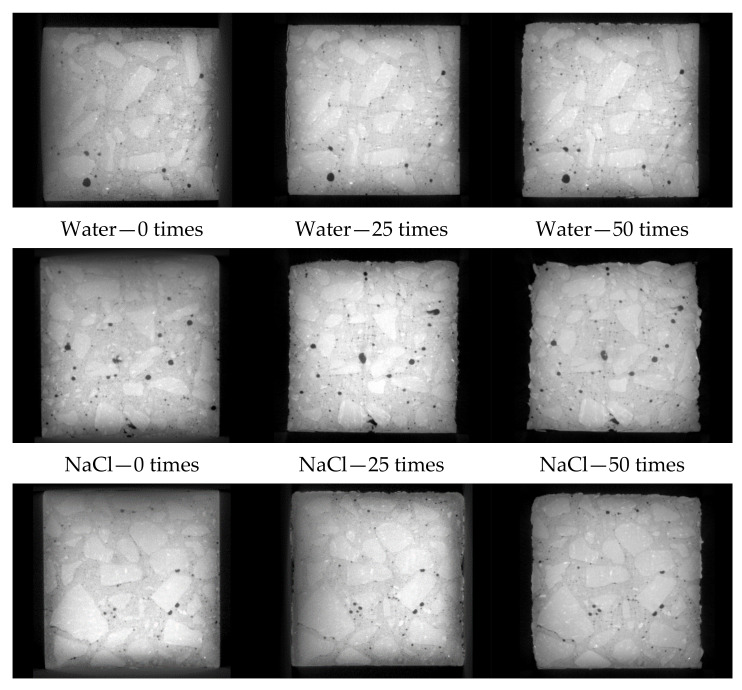

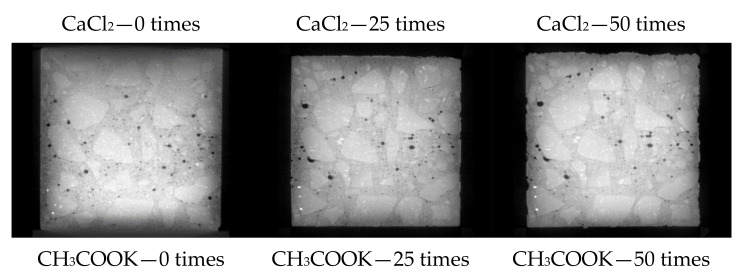
ICT slice images of concrete inter-layer under different salt freezing environment.

Considering the 500th slice of the intermediate layer as an example (Figure 4), the concrete surface is flat and regular before freezing and thawing. After 25 freezing and thawing cycles, the surface is serrated, a few cracks appear at the edge, mortar peeling appears on some surfaces, and small holes appear in the concrete. After 50 freeze-thaw cycles, the number of spalling increases, the spalling phenomenon is more obvious, and the internal pores increase. Some small- and medium-sized pores gradually evolve into macropores during the freeze-thaw cycle. In the clean water and CH_3_COOK solution environments, the surface of the test piece is still relatively complete after 50 freeze-thaw cycles; only part of the mortar is peeled off, in a dispersed state, and the aggregate is slightly exposed. After 50 freeze-thaw cycles in the CaCl_2_ solution, although passivation occurs at the edges and corners, its shape is relatively regular. A small amount of aggregate is exposed, and the mortar peeling phenomenon is severe. In the NaCl solution environment, the surface mortar peeling and roughness of the test piece occurred after 25 freeze–thaw cycles. The closed hole in the lower surface of the test piece forms an opening due to mortar peeling. After 50 freeze-thaw cycles, the surface has severe mortar peeling; many aggregates are exposed and severely deteriorate.

**Figure 5:**
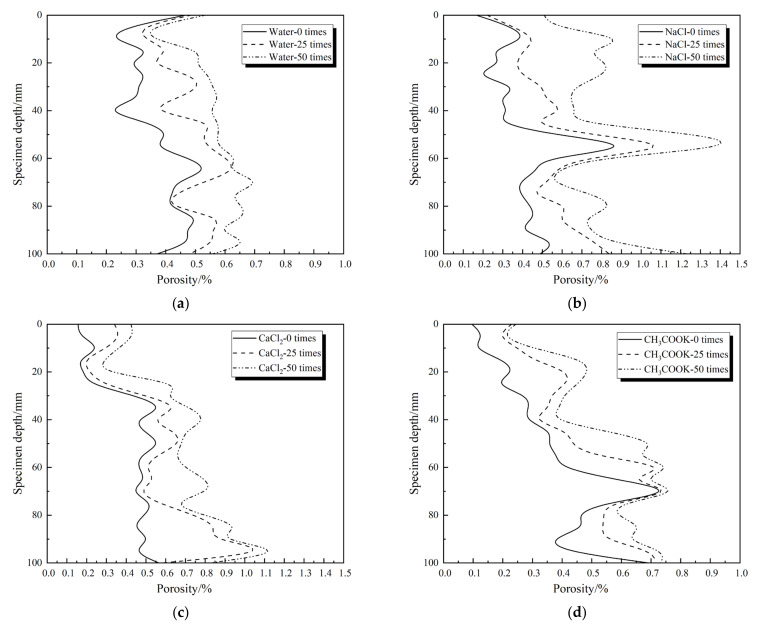
Changes in layered porosity of specimens in different salt freezing environments: (**a**) Water; (**b**) NaCl; (**c**) CaCl_2_; (**d**) CH_3_COOK.

Figure 5 and Table 2 show that as the freezing and thawing times increase, the porosity of the specimen increases from top to bottom along the height direction. Additionally, the internal structure of the concrete deteriorates, and the porosity increases continuously. There are certain differences in the impact of different solution environments on the concrete porosity. The average porosity of the concrete in a clean water environment and CH_3_COOK solution environment changes more evenly. The variance changes slightly, indicating that the pore change trend of each layer of the specimen is relatively stable in the freeze–thaw cycle. In the NaCl solution environment, the 2D porosity of the specimen changes along the pouring depth direction. The porosity significantly fluctuates when it is close to the upper and lower surfaces and the middle of the specimen. The variance change is greater than that of the other three solutions. The greater the variance, the greater the dispersion of the porosity, the uneven porosity of each layer on the pouring surface, and the most obvious deterioration effect on the specimen. The change in the 2D porosity of the specimen in the CaCl_2_ solution environment is mainly concentrated in the lower half. The variance is the second only to the NaCl group, indicating that the deterioration of the specimen is concentrated in the lower half, with the largest change occurring between 80 and 90 mm. The NaCl solution has the greatest impact on concrete deterioration, followed by CaCl_2_. The clean water solution is similar to the CH_3_COOK solution and has little impact on concrete deterioration. This result is consistent with the results of the concrete frost resistance durability test, which further verifies that the change in concrete porosity will affect its performance.

The samples frozen and thawed in the NaCl and CaCl_2_ solution are more severely damaged than those in the clean water and CH_3_COOK solution. The reason for this is that the freezing expansion rate of the NaCl solution is larger than that of the aqueous solution, and the internal pores are more frozen and expanded. The salt freezing damage of concrete frozen and thawed in the CaCl_2_ solution is due to the formation of compound salt, CaCl_2_·Ca(OH_2_)·H_2_O, significantly reducing the frost resistance of concrete, thus causing erosion damage of the concrete [47]. The freeze–thaw damage of the concrete in the inorganic salt CH_3_COOK solution is much smaller than that of the chloride solution. Additionally, the degree of freeze–thaw damage is similar to that of the clean water environment, indicating that the existence of Cl^−^ is the main reason for reducing the frost resistance of concrete.

### 3.3. Three-Dimensional Pore Structure Characteristics

Avizo software is used for threshold segmentation of 2D section data. The pores of each layer of the concrete slice are extracted using different gray levels for 3D reconstruction to obtain the 3D model of internal pores and analyze the influence characteristics of the freeze–thaw environment on concrete pores from the 3D pore distribution. Different pores are classified into large, medium, and small according to the pore volume. Among them, pores with volume V less than 0.1 mm^3^ are small pores, 0.1 mm^3^ ≤ V ≤ 1 mm^3^ are medium pores, and V greater than 1.0 mm^3^ are large pores.

**Figure 6:**
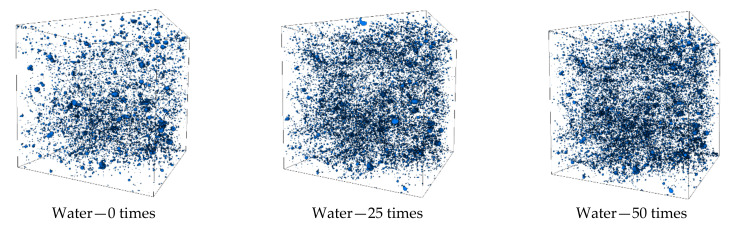

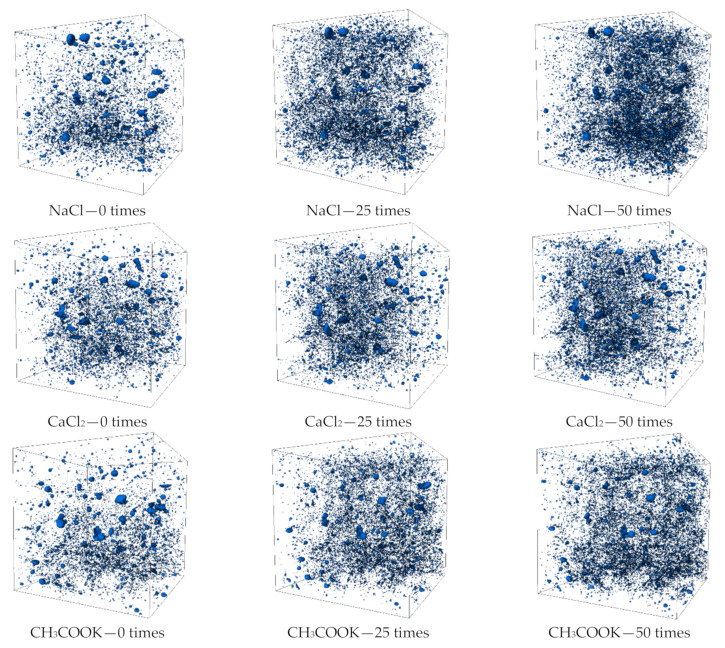
Three-dimensional pore model of concrete in different salt freezing environments.

By analyzing the changes of large, medium, and small holes in concrete, the influence laws of the freeze–thaw environment and cycle on the internal pore structure of concrete are obtained. Figure 6 and Table 3 show that there are many pores in the lower part of the specimen when the concrete is frozen and thawed zero times in the clean water environment. As the number of freeze–thaw cycles increases, additional pores gather in most areas of the specimen. From the pore structure perspective, the volume of medium and large pores accounts for more. After 25 freeze–thaw cycles, the number of medium and small holes doubled. The proportion of the pore volume increased more, whereas the proportion of large pore volume decreased more. Additionally, the porosity increased by 0.1% compared with 0 freeze–thaw cycles. After 50 freezing and thawing times, the growth rate of the number of large, medium, and small pores slowed down; the proportion of the volume of large pores increased, and the proportion of the volume of medium and small pores decreased slightly. However, the porosity still increased by 0.09% compared with 25 freezing and thawing times. The reason is that although the number of pores did not increase much, the pore volume increased gradually.

Figure 6 shows that after freezing and thawing, the internal pores of the test piece in the NaCl solution environment increase, and small pores appear when there are no pores. Some parts of the small pores are connected to form connected pores, and part of the volume expands to form medium and large pores. In the initial state, the proportion of the pore volume of the test piece is mainly large pores. After 25 freeze-thaw cycles, the number of medium and small pores increased several times. From the pore structure perspective, the proportion of large pore volume decreased significantly; the proportion of medium and small pore volume increased, and the porosity increased by 0.16% compared with zero freeze–thaw cycles. After 50 freeze-thaw cycles, the growth trend of the pore number did not slow down, and the total number of pores increased further. Among them, the proportion of large pore volume decreased further; the growth rate of the small pore volume slowed down. The proportion of the medium pore volume is 42.3%, which is the largest, and the total porosity increased by 0.23% compared with 25 freeze-thaw cycles.

In the CaCl_2_ solution environment, when the concrete specimen is frozen and thawed zero times, the volume proportion primarily comprised large pores; the volume proportion of small pores is relatively low. Additionally, most of the pores are concentrated in the middle and lower part of the specimen. After 25 freeze-thaw cycles, many pores were generated on the left side of the specimen. The change in the number of pores is similar to that of other solutions. They increase exponentially, mainly medium and small pores. The change in the proportion of pore volume is small; the proportion of large pores decreases; the increase in the proportion of medium pores is small; the proportion of small pores increases more, mainly small pores; and the porosity increases by 0.14%. After 50 freeze–thaw cycles, the number of medium and small holes increases. The proportion of pore volume changes due to the growth of medium holes, and there is a small decrease in large holes, a small increase in small holes, and the porosity increases by 0.13% compared with 25 freeze–thaw cycles.

In the CH_3_COOK solution environment, when the concrete is frozen and thawed zero times, the proportion of pore volume is mainly macropores, and most of the pores are concentrated in the lower part of the specimen. After 25 freeze–thaw cycles, the growth rate of the pore number is smaller than that of the other three solutions; the total number is less than 20,000; the proportion of macropore volume decreases more; the proportion of mesopore volume increases exponentially; the proportion of small pore volume also increases slightly; and the porosity increases by 0.11% compared with that of zero freeze–thaw cycles. After 50 freeze–thaw cycles, the total number of pores increased by 2059. From the change in the pore volume proportion, the proportion of large pores further decreased; the proportion of medium pores increased more; the proportion of small pore volume increased slightly; and the porosity changed slightly, increasing by 0.07% compared with 25 freeze-thaw cycles.

### 3.4. Effect of Salt Solution on the Pore Structure of Concrete

**Figure 7:**
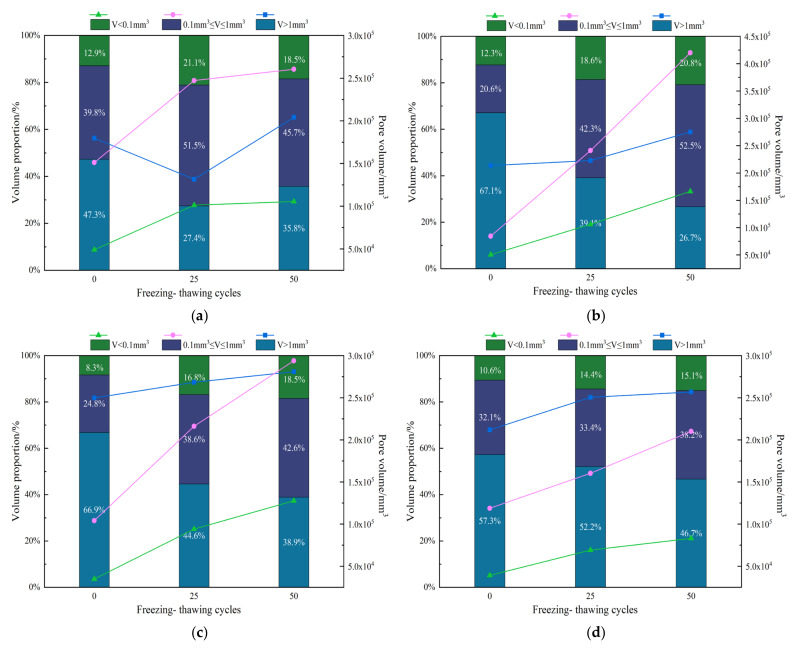
The pore volume and proportion of large, medium, and small pores in concrete under different salt freezing environment: (**a**) Water; (**b**) NaCl; (**c**) CaCl_2_; (**d**) CH_3_COOK.

Different salt freezing environments have different effects on the changes in the internal pores of the concrete. With the increase in the freezing and thawing times, the proportion of pore volume with different sizes will also change. As shown in Figure 7, the proportions of the pore volumes of large, medium, and small holes change in different salt freezing environments after 50 freeze-thaw cycles. The proportion of pore volume less than 0.1 mm3 increases from 12.3 to 20.8%; the proportion of pore volume less than 0.1 mm^3^ ≤ V ≤ 1 mm^3^ increases greatly from 20.6% in the initial state to 52.5%. However, the proportion of pore volume V greater than 1 mm^3^ decreases gradually from 67.1 to 26.7%. This shows that it is mainly dominated by the growth of mesopores during the freezing and thawing of the NaCl solution. The pore growth trend in the CaCl_2_ solution environment is similar to that in the NaCl solution (Figure 7c). However, the overall pore trend is relatively slow. After freezing and thawing, the proportion of the pore volume of small pores increases by 10.2%, and the proportion of the pore volume of medium pores increases by 17.8%. In the CH_3_COOK solution environment, the overall proportion of pore volume slowed down again; the proportion of pore volume increased by 4.5%; and the proportion of mesoporous pore volume increased by 6.1%. Figure 7a shows that the change in the internal pores of the freeze–thaw concrete fluctuates in the clean water environment. Additionally, the proportion of medium and small pore volume decreases first and then increases, and the proportion of large pore volume decreases first and then increases.

Therefore, it can be seen that the effect of salt solution on the internal pore structure of concrete is consistent with the effect of salt solution on the frost resistance durability of concrete. The influence trend of salt solution on pores is that the proportion of medium and small pore volume increases, and the proportion of large pore volume decreases. However, freezing and thawing in chloride solution environment accelerate the change in the pore structure of concrete, resulting in severe salt freezing damage of concrete. Comparing the pore structure changes of concrete in salt solution and clean water environments, it can be concluded that the change of mesopore pore volume is the main factor of concrete salt freezing damage. Therefore, the greater the changing trend, the more severe the damage caused by salt freezing.

### 3.5. Effect of Pore Change on the Compressive Strength of Concrete

Changes in porosity may affect the performance of concrete. To study the relationship between the concrete compressive strength and porosity, the least square method is used to linearly fit the change in concrete strength with the increase in porosity after the freeze–thaw cycle in four environments. The fitting equation is as follows:

(4) $$P=a+bf_{cu,k}$$

(5) $$R^{2}=0.82391$$

In relations (4) and (5), *P* is the overall porosity of concrete, *f_cu_*_,*k*_ (MPa) is the cube compressive strength of concrete, *a* and *b* are constants (*a* = 35.04, *b* = −16.63), and the value is determined by the test, where *R*^2^ is the error.

**Figure 8:**
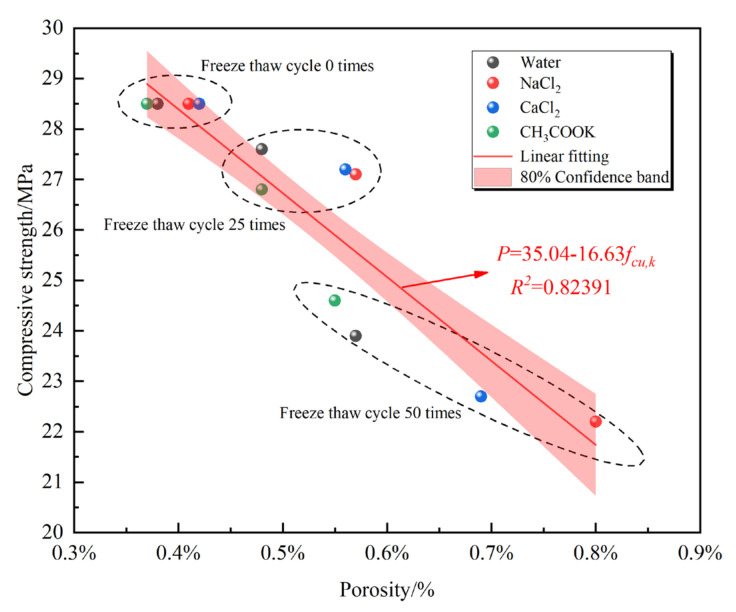
Relationship between porosity and compressive strength.

The fitting curve shows that the porosity is negatively correlated with the compressive strength of concrete. As the porosity increases, the compressive strength of concrete decreases. Figure 8 shows that the porosity of concrete in different environments shows an upward trend as the number of freeze–thaw cycles increases. Freezing and thawing in chloride solution have a significant impact on the porosity of concrete. The non-chloride salt and clean water environments have little effect on the overall porosity of concrete. This change is consistent with the durability test results. By analyzing the test data and fitting curve, it can be obtained that in any environment, as long as the concrete is in the state of the freeze–thaw cycle, the porosity of the concrete will change. The change in porosity affects the compressive strength of concrete. Thus, the greater the change in porosity, the smaller the compressive strength of concrete. The reason is that when the concrete is frozen in the water-saturated state, the volume of water in the pores freezes and expands. The cementitious pores have osmotic pressure on the pores, further expanding the ice volume in the pores. When the expansion and osmotic pressure exceed the concrete limit, the concrete will be damaged, and its strength will be gradually reduced.

## 4. Conclusions

Based on ICT scanning technology, this paper analyzes the frost resistance durability and pore characteristics of concrete in the salt freezing environment. The following conclusions are obtained through concrete durability and compressive strength tests.

In any solution environment, with the increase in freeze-thaw cycles, the concrete will have a loss in compressive strength, an increase in mass loss rate, and a decrease in the relative dynamic elastic modulus. Chloride deicing salt 5% NaCl and 5% CaCl_2_ solution have the greatest impact on the frost resistance durability and mechanical properties of concrete. The impact of non-chloride deicing salt 5% CH_3_COOK on concrete is similar to that of the clean water solution. It has little impact on the frost resistance durability and mechanical properties of concrete. It means that the freeze–thaw damage of concrete in the chloride deicing salt environment is more severe than that in the non-chloride deicing salt and clean water environments. Therefore, when deicing ordinary concrete, it is recommended to give priority to non-chloride deicing salts.

With an increase in freeze–thaw cycles, the porosity of concrete in different environments shows an upward trend. After the freeze–thaw cycles, the increase in the internal porosity of concrete from high to low is 5% NaCl, 5% CaCl_2_, clean water, and 5% CH_3_COOK. In any solution environment, the compressive strength of concrete is negatively correlated with porosity. It means that the greater the porosity, the lower the compressive strength of the concrete. The test is aimed at ordinary concrete, and the use of concrete with special functions needs to be studied, such as the use of an air entraining agent to improve the thermal insulation function of concrete.

The influence of different salt solutions on concrete stays on the surface and has a certain impact on the internal pores of the concrete. The internal pores of chloride concrete change significantly, which is reflected in the variance of 2D porosity. The larger the variance, the greater the influence of salt solution on the 2D pore characteristics of concrete. The influence of the salt solution on the 2D pore characteristics of concrete from largest to smallest is 5% NaCl, 5% CaCl_2_, clean water, and 5% CH_3_COOK. The use of non-chlorine salt deicing agents can reduce the impact of freezing damage on concrete to a certain extent.

Under the salt solution environment, freezing and thawing greatly influence the internal pores of the concrete. The proportion of large pore volume decreases, and the proportion of medium and small pore volumes increase. Among them, the faster the growth rate of the proportion of the medium pore volume, the greater the impact on the frost resistance durability and mechanical properties of concrete. The increase in the proportion of the medium pore volume from the largest to smallest increase is 5% NaCl, 5% CaCl_2_, and 5% CH_3_COOK. It is consistent with the experimental results of durability and mechanical properties. This test analyzes the internal pore changes of concrete and obtains the pore change trend of different salt solutions. This change is the same as the change trend of the durability test. It aims to study the change law of the internal pores. There are certain limitations, but the pore pair can be changed for further research. The impact of concrete performance provides a research basis.

## Figures and Tables

**Table 1:** Mix proportions of concrete.

Scheme	Cement/kg	Fly Ash/kg	Sand/kg	Stone/kg	Water/kg	Additive/kg
C30	270	90	854	1004	165	6.3

**Table 2:** Average porosity and variance of concrete.

Serial Number	W^1^-0	W-25	W-50	N^2^-0	N-25	N-50	C^3^-0	C-25	C-50	K^4^-0	K-25	K-50
**Average porosity/%**	0.38	0.48	0.57	0.41	0.57	0.80	0.42	0.56	0.69	0.37	0.48	0.55
**Variance**	0.010	0.009	0.009	0.034	0.047	0.071	0.021	0.059	0.056	0.033	0.032	0.030

W^1^ represents clean water; N^2^ represents NaCl; C^3^ represents CaCl_2_; K^4^ represents CH_3_COOK; N represents freezing and thawing times.

**Table 3:** Number of large, medium, and small pores and overall porosity of concrete before and after freezing and thawing.

Environment	Times of Freezing–Thawing	Volume Type, Quantity, and Volume Proportion of Pores						Numbers of Pores	Porosity/%
		V > 1 mm^3^	Volume Proportion	0.1 mm^3^ ≤ V ≤ 1 mm^3^	Volume Proportion	V < 0.1 mm^3^	Volume Proportion		
Water	0	193	47.3%	2554	39.8%	7950	12.9%	10,697	0.38
	25	219	27.4%	4345	51.5%	16,785	21.1%	21,349	0.48
	50	232	35.8%	4637	45.7%	18,578	18.5%	23,447	0.57
NaCl	0	171	67.1%	2426	20.6%	7322	12.3%	9919	0.41
	25	195	39.1%	4608	42.3%	20152	18.6%	24,955	0.57
	50	211	26.7%	5456	52.5%	25,831	20.8%	31,498	0.80
CaCl_2_	0	126	66.9%	2709	24.8%	9301	8.3%	12,136	0.42
	25	145	44.6%	4211	38.6%	17825	16.8%	22,181	0.56
	50	157	38.9%	5186	42.6%	21,028	18.5%	26,371	0.69
CH_3_COOK	0	205	57.3%	2763	32.1%	8584	10.6%	11,552	0.37
	25	209	52.2%	3779	33.4%	15,248	14.4%	19,236	0.48
	50	220	46.7%	4443	38.2%	16,632	15.1%	21,295	0.55

## Data Availability

Not applicable.
